# Altered striatal dopamine regulation in *Adgrl3* knockout mice

**DOI:** 10.1038/s41386-026-02474-3

**Published:** 2026-06-26

**Authors:** Nicole A. Perry-Hauser, Arturo Torres-Herraez, Siham Boumhaouad, Emily A. Makowicz, Danielle C. Lowes, Michelle Jin, Christine A. Denny, David Sulzer, Eugene V. Mosharov, Christoph Kellendonk, Jonathan A. Javitch

**Affiliations:** 1 Department of Psychiatry, Columbia University Vagelos College of Physicians and Surgeons, New York, NY USA; 2 Department of Molecular Pharmacology and Therapeutics, Columbia University Vagelos College of Physicians and Surgeons, New York, NY USA; 3 Division of Molecular Therapeutics, New York State Psychiatric Institute, New York, NY USA; 4 Department of Neurology, Columbia University Irving Medical Center, New York, NY USA; 5 Physiology and Physiopathology Team, Genomics of Human Pathologies Research Center, Faculty of Sciences, Mohammed V University in Rabat, Rabat, Morocco; 6 Division of Systems Neuroscience, New York State Psychiatric Institute, New York, NY USA; 7 Columbia University’s Medical Scientist Training Program (MSTP), New York, NY USA; 8 Present Address: Centre for Translational Pharmacology, School of Molecular Biosciences, University of Glasgow, Glasgow, UK

**Keywords:** Reward, Motivation, Electrophysiology, Optical spectroscopy

## Abstract

Dopamine signaling is essential for regulating movement, learning, and reward processing, and its dysregulation has been implicated in neuropsychiatric conditions such as ADHD and substance use disorder. ADGRL3, an adhesion G protein-coupled receptor enriched in the brain, has been genetically linked to these disorders, and *Adgrl3* knockout in animals alters expression of dopaminergic markers and impacts dopamine-related behaviors. However, how ADGRL3 influences dopamine dynamics remains poorly understood. Here, we characterized striatal dopamine release in *Adgrl3* knockout mice using complementary ex vivo and in vivo approaches. Fast-scan cyclic voltammetry in acute brain slices revealed increased electrically evoked dopamine release in both dorsal and ventral striatum of *Adgrl3* knockout mice. In contrast, in vivo fiber photometry using the dopamine sensor dLight1.2 showed reduced cue-induced dopamine signals in the ventral striatum during an operant fixed interval task. This reduction was accompanied by longer latencies to lever press and retrieve rewards, consistent with altered cue-guided behavioral responses reminiscent of task-switching difficulties in individuals with ADHD. Amphetamine challenge experiments indicated that phasic release capacity was similar between genotypes, suggesting that dopamine stores are intact in *Adgrl3* KO mice. Together, these findings reveal that ADGRL3 regulates striatal dopamine release and motivate mechanistic studies of how its loss may alter the spatial organization of dopaminergic terminals.

## Introduction

*Adgrl3* (also known as *Lphn3*) encodes an adhesion G protein-coupled receptor (aGPCR) of the latrophilin subfamily. It is expressed broadly in the nervous system, with levels peaking early postnatally and gradually declining to adult levels during maturation [1, 2]. At the synaptic level, ADGRL3 contributes to hippocampal synapse formation through trans-synaptic interactions with teneurins and fibronectin leucine-rich repeat transmembrane proteins (FLRTs), supporting roles in synaptic plasticity and development [3–9]. In humans, genetic studies link polymorphisms in *ADGRL3* to an increased risk for attention-deficit/hyperactivity disorder (ADHD) and susceptibility to substance use disorders [1, 10].

Alterations in dopamine signaling are hypothesized to contribute to ADHD pathophysiology [11], and *Adgrl3* knockdown or knockout across species disrupts dopamine signaling and leads to abnormalities in dopamine-related behaviors, most notably increased locomotor activity [12–18]. Previous molecular and neurochemical characterizations of *Adgrl3* knockout (KO) in rodents identified altered expression of dopamine-related genes (e.g., *Dat1, Drd4, Th*) and proteins (e.g., TH, AADC, DAT, DARPP-32), with mixed results regarding striatal dopamine levels at the biochemical level [16, 17]. Collectively, these observations implicate ADGRL3 in the regulation of striatal dopamine function; however, how ADGRL3 influences dopamine dynamics during behavior remains unknown.

Dopamine plays a critical role in movement, learning, and motivation [19, 20], and *Adgrl3* KO rodents show broad behavioral differences spanning motor, cognitive, and motivational domains. Motor assessments, including rotarod, open field, and gait analysis, show hyperactivity and unstable footing compared to wild-type (WT) animals [14, 16]. Learning and memory deficits include impaired object discrimination in a novel object recognition task and reduced visuospatial memory in the Barnes maze [14]. Motivational and reward-related differences have been identified across several tasks: increased lever pressing in a fixed ratio schedule of reinforcement [15], delayed immobility in the forced swim test [14], and heightened impulsivity with slower reward retrieval in the continuous performance test [14]. Overall, *Adgrl3* deletion produces behavioral abnormalities that have been interpreted as reflecting hyperdopaminergic function; yet, whether and in what contexts dopamine release dynamics are altered during behavior has not been directly established.

Here, we provide an integrated behavioral and neurochemical characterization that establishes a foundation for understanding how loss of *Adgrl3* shapes striatal dopamine function. We first confirm increased locomotor activity in *Adgrl3* KO mice using open field testing and home-cage monitoring. We then pair two complementary readouts of dopamine release: ex vivo fast-scan cyclic voltammetry (FSCV) in acute striatal slices and in vivo fiber photometry using the genetically encoded dopamine sensor dLight1.2 during a fixed-interval schedule. While FSCV revealed elevated dopamine release in slices, in vivo cue-evoked dopamine during fixed interval behavior was reduced in KO mice; moreover, amphetamine produced comparable dopamine responses across genotypes.

Our findings show a divergence: ex vivo measurements predicted a hyperdopaminergic phenotype, yet cue-evoked dopamine is blunted in KO mice during behavior. These results establish a foundation for mechanistic studies of how ADGRL3 shapes striatal dopamine function.

## Materials and methods

Complete methodological details for all experiments are provided in the Supplement.

### Mice

Adult *Adgrl3* knockout mice and WT controls derived from the same breeding colony (MGI:4973916) were group-housed under a 12-h light/dark cycle (22°C) with *ad libitum* access to food and water. Genetic monitoring (Transnetyx miniMUGA) confirmed a predominantly C57BL/6J and C57BL/6NCrl background with <10% 129S4/SvJaeJ contribution. Because homozygous KO breeding was unreliable (see “Discussion”), some WT controls were not littermate-matched but were maintained on the same verified background. All procedures were approved by Columbia University and NYSPI IACUCs in accordance with NIH guidelines.

### Open field

Locomotor activity was assessed for 60 min in a photobeam-equipped plexiglass enclosure (Med Associates), quantifying horizontal movement, rearing, and center vs. periphery time.

### PiezoSleep study

Sleep/wake behavior was recorded for five days using the PiezoSleep system (Signal Solutions LLC) following established protocols [21, 22].

### Gustometer

Reward sampling behavior was assessed using a gustometer (Med Associates) under mild water restriction.

### Fast-scan cyclic voltammetry (FSCV)

Striatal dopamine release was measured in acute coronal slices using standard FSCV approaches [23].

### Stereotaxic surgery and fiber photometry

Mice received unilateral AAV-hSyn-dLight1.2 injections and optic fiber implants targeting the nucleus accumbens (AP + 1.7 mm, ML ± 1.2 mm, DV, -4.2, -4.1, and -4.0 mm; 125 nL/site). Dual wavelength photometry (405/465 nm) was used to record dLight1.2 signals during behavior.

### Operant behavior

Mice underwent dipper training, continuous reinforcement (CRF), and fixed interval (FI: 2-24 s) schedules using standard operant chambers (Med Associates).

### Amphetamine challenge

A subset of photometry-implanted mice received saline and increasing doses of amphetamine (1.5–20 mg/kg, s.c.) in a 60-min open field test to assess dopamine release capacity (adapted from [24]).

### Statistical analysis

Analyses were performed using GraphPad Prism or MATLAB, with specific tests and n values provided in figure legends. Sample sizes varied across some experiments due to fiber loss in chronic implants and a priori exclusion criteria. Both sexes were included; sex-stratified analyses for open field behavior appear in Fig. S1, but the study was not powered to formally test for sex differences across all measures.

## Results

### *Adgrl3* KO mice exhibit increased locomotor activity across development

**Fig. 1:**
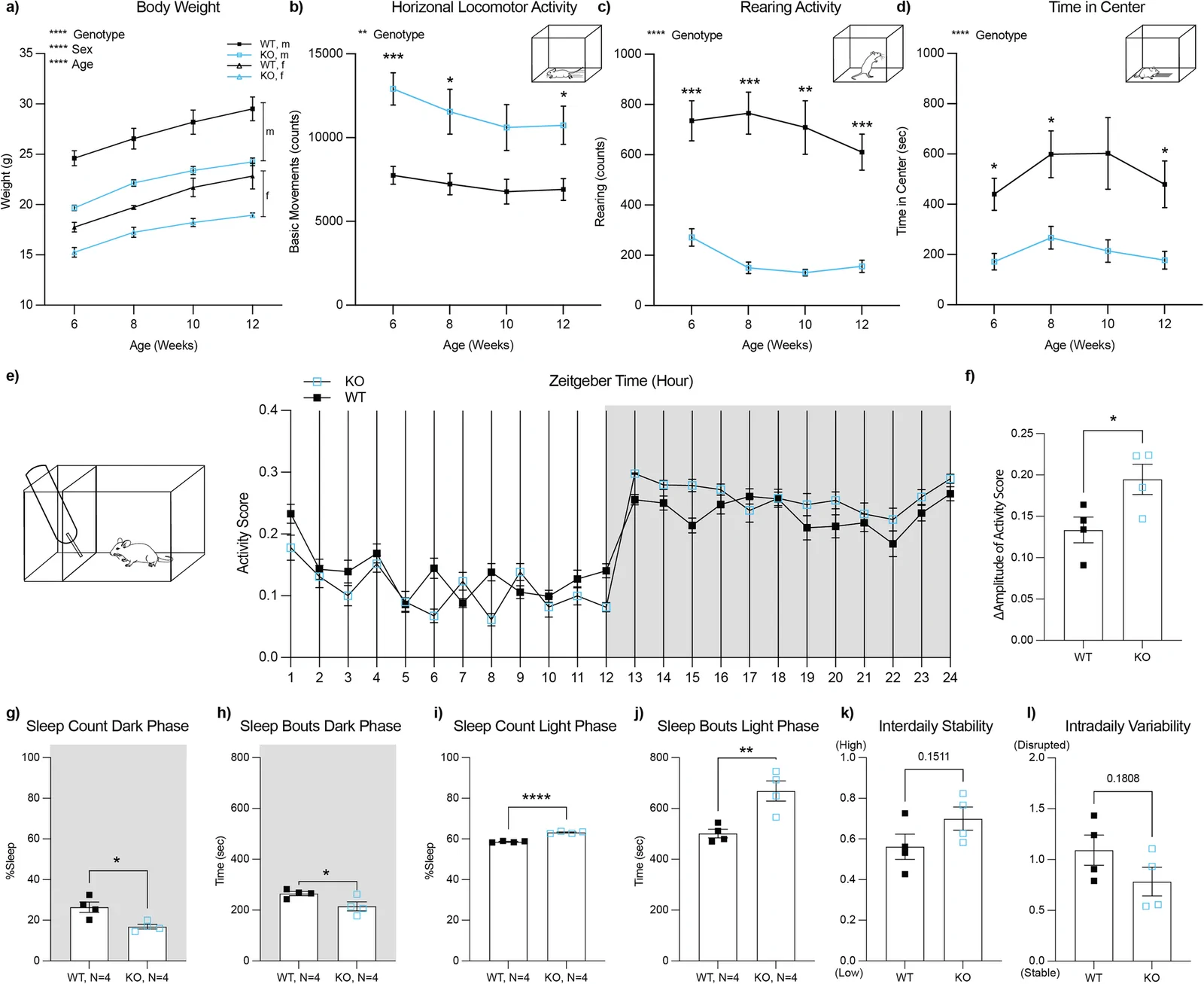
*Adgrl3* knockout mice show increased activity and altered sleep patterns. **a** Body weight of wild-type (WT) and *Adgrl3* knockout (KO) mice of both sexes (WT: *n* = 9; 5 males, 4 females; KO: *n* = 17, 13 males, 4 females). Three-way ANOVA was performed to analyze the effect of age, sex, and genotype on body weight (****, *p* < 0.0001). **b–d** Horizontal locomotor activity, rearing, and center time across development. Two-way repeated measures ANOVA was performed to analyze the effect of age and genotype on each dependent variable (basic movements, rearing, or time in center). This was followed by Ṥídák’s multiple comparisons test (*, *p* < 0.05; **, *p* < 0.01; ***, *p* < 0.001; ****, *p* < 0.0001). **e**, **f** PiezoSleep system activity scores and amplitude across Zeitgeber time (WT: *n* = 4; 1 male, 3 females; KO: *n* = 4, 1 male, 3 females). **g–j** Sleep metrics during dark and light cycles: percent time asleep and average bout duration. **k, l** Interdaily stability and intradaily variability quantify rhythm regularity and fragmentation [49]. Unpaired t-tests used for all PiezoSleep comparisons (****, *p* < 0.0001; ***, *p* < 0.001; **, *p* < 0.01; and *, *p* < 0.05). Illustrations used in this image were adapted from SciDraw.

Compared to WT controls, *Adgrl3* KO mice weighed less (Figs. 1a and  S1A, E) and travelled greater horizontal distances along both X and Y axes in the open field test (Figs. 1b and  S1B, F; S2A, B), indicating increased baseline locomotor activity, consistent with previous reports in this model [14, 16]. They also showed reduced rearing behavior (Figs. 1c and  S1C, G) and spent less time in the center of the enclosure (Figs. 1d and  S1D, H), a pattern confirmed by spatial distribution analysis (Fig. S2C, D). While reduced center time and rearing are often interpreted as anxiety-like behavior [25], these measures may be confounded by increased activity in *Adgrl3* KO mice.

### *Adgrl3* knockout mice exhibit increased activity during the dark cycle compared to controls

To assess whether elevated activity in *Adgrl3* KO mice varies by time of day, we monitored sleep/wake behavior using the PiezoSleep system over five days under a 12-hour light/dark cycle. *Adgrl3* KO mice showed elevated activity across the light-dark transition (Fig. 1e, f), likely driven by reduced sleep and shorter sleep bouts during the dark (active) phase (Fig. 1g, h). This is consistent with prior findings in *Adgrl3* KO rats [17]. During the light cycle, *Adgrl3* KO mice slept more and had longer sleep bouts (Fig. 1i, j), suggesting an altered sleep-wake regulation. We also analyzed interdaily stability, a measure of daily consistency, and intradaily variability, which reflects fragmentation of activity within a 24-hour period (Fig. 1k, l). Neither measure differed significantly between genotypes.

### *Adgrl3* KO mice show increased licking behavior for evaporated milk

To assess hedonic drive, WT and *Adgrl3* KO mice were tested in a six-bottle preference task using a gustometer (Fig. S3) [26]. While previous studies in *Adgrl3* KO mice have examined motivational responses to food rewards, the hedonic (“liking”) component has not been directly assessed. Mice sampled water and five concentrations of evaporated milk (6.25–100%) over 30 min. *Adgrl3* KO mice completed more trials, initiated licking more frequently, and showed shorter latencies to first lick compared to WT controls (Fig. S3B–E), suggesting enhanced reward engagement. However, when analysis was restricted to completed trials, the average lick count per solution did not differ between genotypes (Fig. S3F–H), suggesting that the elevated responding in Adgrl3 KO mice reflects increased instrumental engagement rather than enhanced hedonic “liking” per se. Fully dissociating these components will require additional paradigms, such as consumption measures under conditions that minimize instrumental demand, and remains a question for future work.

### *Adgrl3* KO mice have greater levels of evoked dopamine release in the striatum

**Fig. 2:**
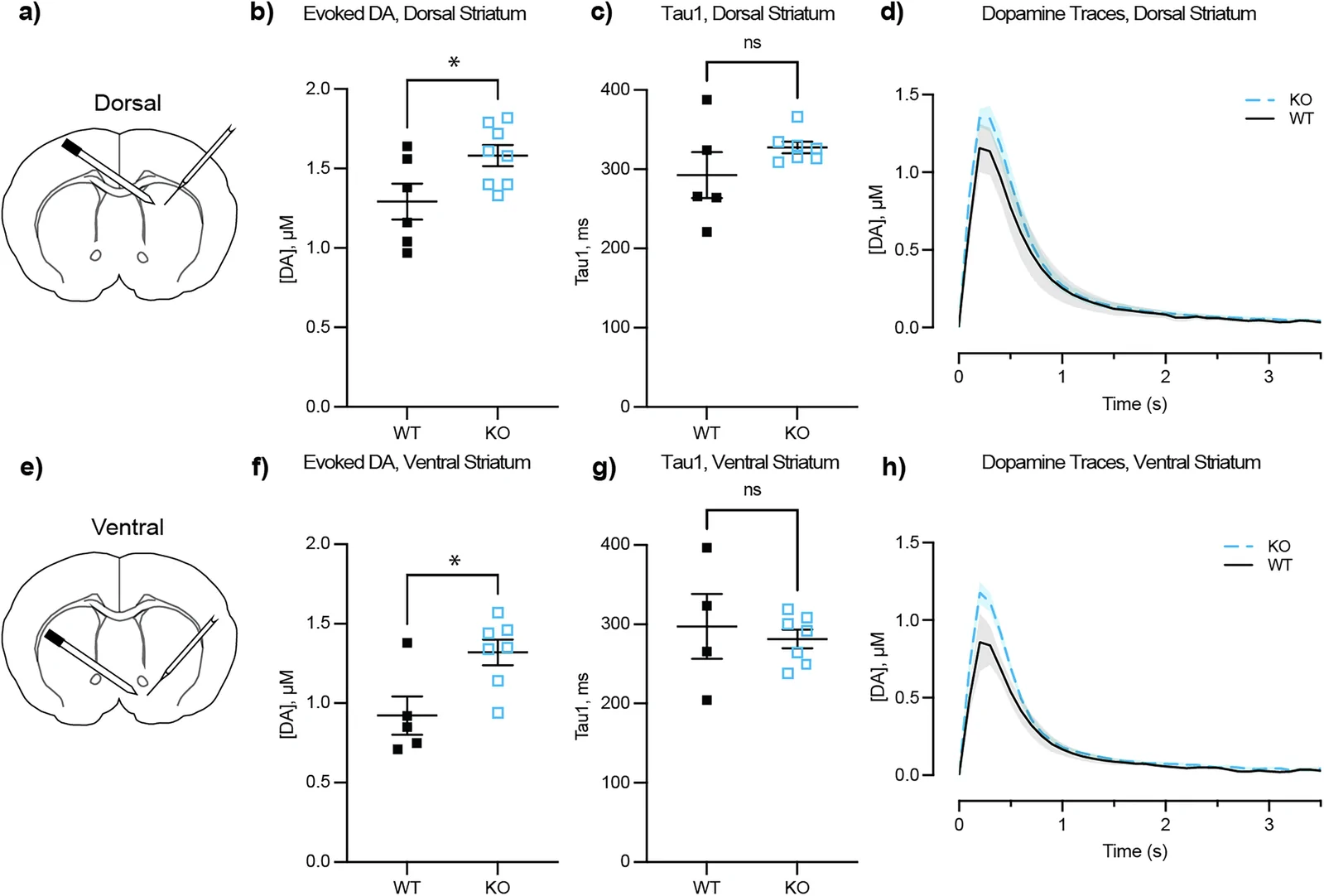
*Adgrl3* KO mice have greater levels of evoked dopamine (DA) in the striatum. Coronal sections that included striatum were obtained from wild-type (WT) and *Adgrl3* knockout (KO) mice aged P102-P183. **a** Schematic of location for fast-scan cyclic voltammetry (FSCV) recordings from the dorsal striatum. Slices were stimulated with a bipolar concentric electrode and DA was detected using a carbon fiber electrode. **b** FSCV recordings of evoked DA release from the dorsal striatum (*N* = 6 WT, *N* = 8 KO; 31 slices WT, 44 slices KO). **c** DA reuptake from the dorsal striatum (*N* = 5 WT, *N* = 7 KO; 25 slices WT, 47 slices KO). **d** Averaged dopamine transients in response to a single electrical pulse in the dorsal striatum from all tested mice, illustrating signal kinetics. **e** Schematic of location for FSCV recordings from the ventral striatum. **f** FSCV recordings of evoked DA release from the ventral striatum (*N* = 5 WT, *N* = 7 KO; 24 slices WT, 36 slices KO). **g** DA reuptake from the ventral striatum (*N* = 4 WT, *N* = 7 KO; 19 slices WT, 42 slices KO). **h** Averaged dopamine transients in response to a single electrical pulse in the ventral striatum from all tested mice, illustrating signal kinetics. All quantal release events were analyzed in IgorPro using a publicly available GitHub-based analysis program [50]. Peak parameters included amplitude (Imax, pA) and event duration (Tau1, ms). All statistical analyses were performed using an unpaired t-test (*, *p* < 0.05). Illustrations used in this image were adapted from SciDraw.

We used Fast-Scan Cyclic Voltammetry (FSCV) to detect electrically evoked dopamine with sub-second precision in brain slices of the dorsal and ventral striatum of adult mice [27] (Fig. 2). Compared to WT controls, *Adgrl3* KO mice showed significantly higher levels of evoked dopamine release in both the dorsal (Fig. 2b, d) and ventral striatum (Fig. 2f, h). Despite the elevated response, there were no significant genotype differences in dopamine reuptake in either region, as indicated by the rate of dopamine signal decay (Tau1) following stimulation (Fig. 2c, g).

### Dopamine release during a continuous reinforcement task is not significantly altered in *Adgrl3* KO mice

**Fig. 3:**
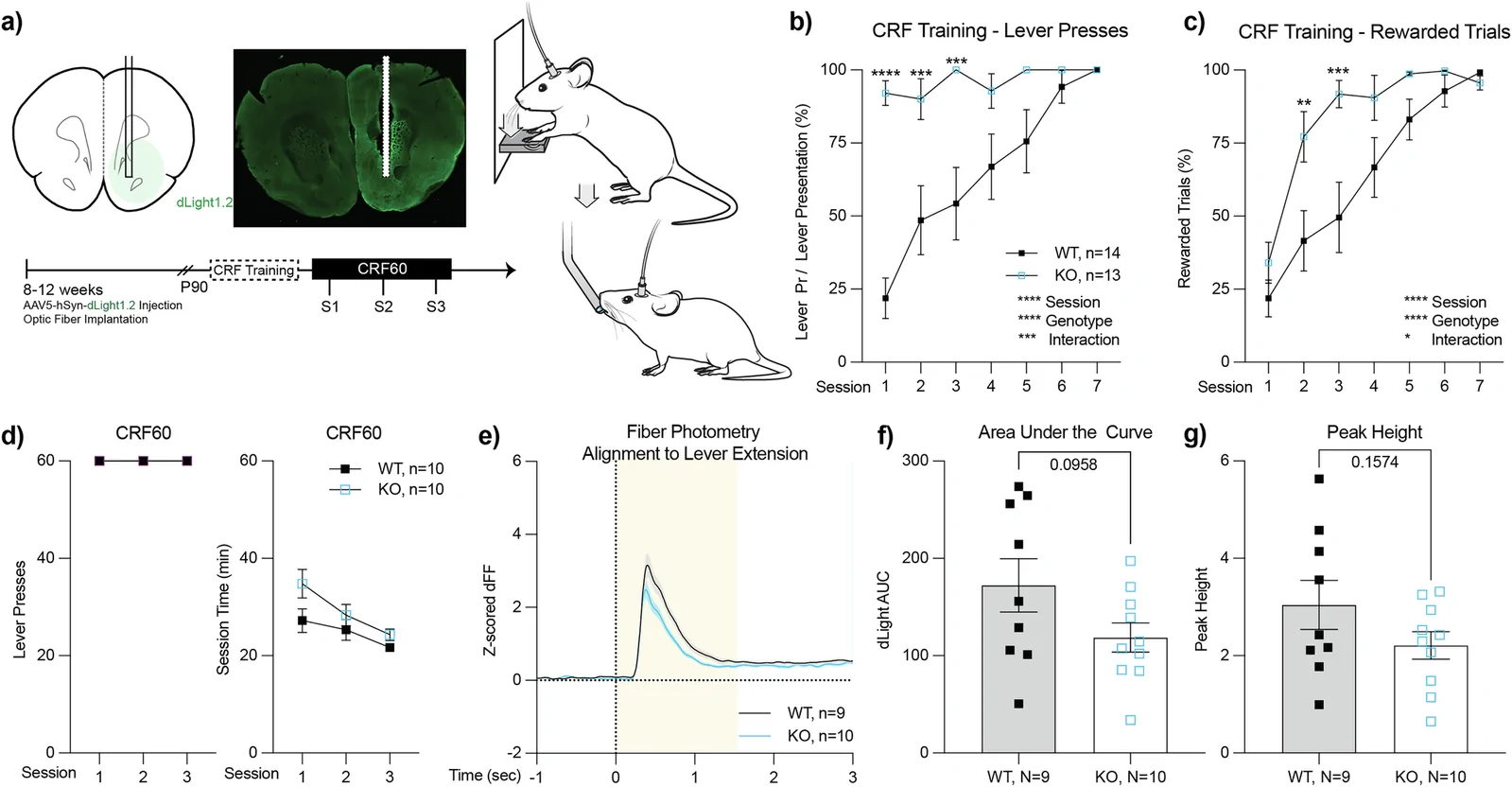
Performance of WT and *Adgrl3* KO mice in a continuous reinforcement task (CRF). **a** (Left) Experimental timeline illustrating progression from viral injection and optic fiber implantation to fiber photometry. (Middle) Optic fibers were unilaterally implanted into the nucleus accumbens. A representative coronal section shows dLight1.2 expression in the striatum and the location of the optic fiber, with dashed lines indicating the fiber shaft. (Right) WT and *Adgrl3* knockout (KO) mice were trained on a fixed-ratio 1 schedule of reinforcement, in which each lever press was rewarded with evaporated milk from a dipper. Training occurred over seven sessions on separate days. **b** Percentage of lever presses relative to total possible lever presentations. **c** Percentage of trials in which mice lever pressed and consumed the evaporated milk reward. Statistical analysis in (**b**) and **c** consisted of a two-way ANOVA followed by Ṥídák’s multiple comparison test (*, *p* < 0.05; **, *p* < 0.01; ***, *p* < 0.001; ****, *p* < 0.0001). (*N* = 14 WT, 13 KO). **d** Following training, WT and *Adgrl3* KO mice completed a continuous reinforcement task where they could earn up to 60 rewards per session. Dopamine levels were monitored via fiber photometry during this operant task. **e** Average dLight1.2 traces aligned to lever extension for WT and *Adgrl3* KO mice. Shaded area (yellow) indicates the timeframe used for AUC calculation. **f** Area under the curve (AUC) and **g** peak height for the dLight1.2 traces. Statistical analysis consisted of an unpaired t test. (*N* = 9 WT, 10 KO). Note: Some animals were excluded from photometry due to inadequate fiber placement or insufficient signal. Illustrations used in this image were adapted from SciDraw.

In the nucleus accumbens, dopamine is released in response to unexpected rewards [20, 28]. With training, this signal shifts to predictive cues. We hypothesized that *Adgrl3* KO mice would exhibit enhanced cue-induced dopamine release based on prior studies in brain slices [18] and our own ex vivo FSCV data. To test this, we used in vivo fiber photometry with dLight1.2 [29] during a continuous reinforcement (CRF) task (Fig. 3).

After viral injection and fiber implantation (Fig. S4), mice were trained to retrieve evaporated milk rewards from a dipper port (30 “free” presentations per session). We found no significant difference in the percentage of rewards retrieved or the total number of rewarded and non-reward head entries between WT and *Adgrl3* KO mice (Fig. S5).

Mice were then trained on a lever-pressing task with a fixed-ratio 1 schedule, where each lever press triggered a reward. *Adgrl3* KO mice acquired lever pressing behavior more quickly than WT mice, completing ~90% of trials in the first session compared to ~22% in controls (Fig. 3b). To determine whether KO mice associated lever pressing with receiving the evaporated milk reward, we measured the percentage of trials in which mice both pressed the lever and consumed the reward (Fig. 3c). In the first session, WT and KO mice had the same percentage of rewarded trials, indicating that despite engaging in more lever pressing, *Adgrl3* KO mice did not exhibit a stronger association between lever pressing and reward. By session four, *Adgrl3* KO mice reached near-perfect performance, indicating faster acquisition of the cue-reward association compared to WT mice, which required seven sessions to reach similar levels.

To further assess learning dynamics, we analyzed lever press and reward latencies across training sessions. *Adgrl3* KO mice pressed the lever significantly faster in early sessions, but when matched by performance level ( ≥ 50% trial completion), latencies were comparable across genotypes. Both groups improved over time, reaching ~5 s for lever press latency and ~1 second for reward retrieval latency (Fig. S6A, B).

Following training, mice completed three CRF sessions where they could earn up to 60 rewards per session. Both genotypes completed all trials and reduced session duration over time (Fig. 3d). During these sessions, dopamine release aligned to lever extension was measured using dLight1.2. No statistically significant differences were observed in either the area under the curve (AUC) or peak amplitude between genotypes (Fig. 3e–g).

Finally, we examined the relationship between dopamine release and lever press latency. Across genotypes, greater dopamine release was associated with faster lever pressing, and correlation strength did not differ between groups (Fig. S7).

### Fixed interval task reveals longer latencies and reduced dopamine release in *Adgrl3* knockout mice

**Fig. 4:**
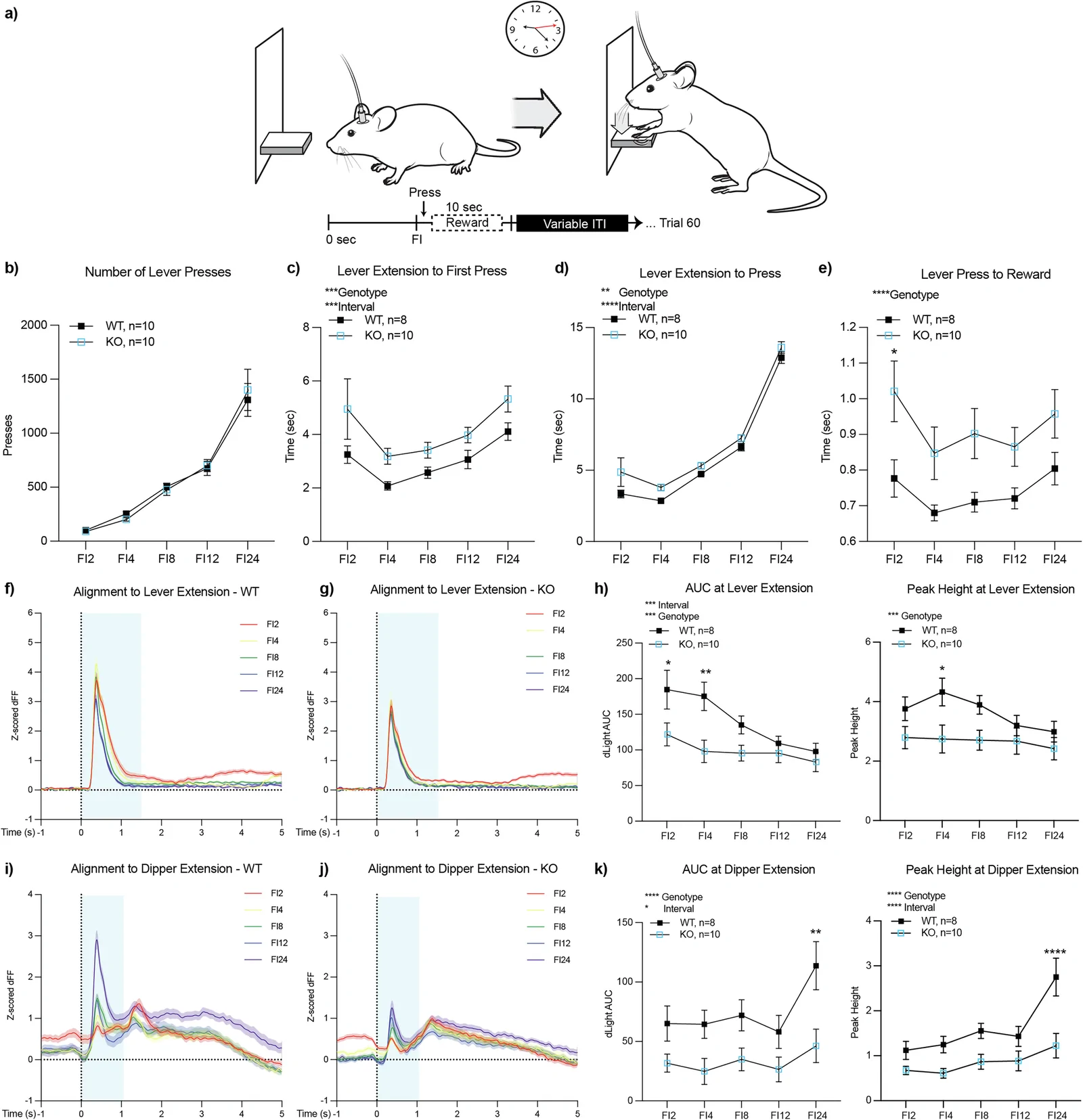
Performance of WT and *Adgrl3* KO mice in a fixed interval task. **a** Mice were tested across five fixed intervals (2-24 s), with three sessions per interval. During each trial, the lever was extended for the designated interval, after which it remained extended until pressed. A successful press delivered a 10-second evaporated milk reward. Each session included up to 60 rewarded trials, with a variable intertrial interval. **b** Total lever presses per interval. No significant genotype differences. (*N* = 10 WT, 10 KO). **c** Latency to first lever press, **d** average latency to lever press, and **e** latency to reward across sessions. All statistical analyses were performed using a two-way ANOVA with Ṥídák’s multiple comparison test (**, *p* < 0.01; ***, *p* < 0.001; ****, *p* < 0.0001). (*N* = 8 WT, 10 KO). **f, g** Average dLight1.2 traces aligned to lever extension for WT and *Adgrl3* KO mice. **h** Area under the curve (AUC) and peak height from 0-1.5 s. Statistical analysis was performed using a two-way ANOVA with Ṥídák’s multiple comparison test (*, *p* < 0.05; **, *p* < 0.01; ***, *p* < 0.001). (*N* = 8 WT, 10 KO). **i, j** Average dLight1.2 traces aligned to dipper extension for WT and *Adgrl3* KO mice. **k** AUC and peak height from 0-1.0 s. Statistical analysis was performed using a two-way ANOVA followed by Ṥídák’s multiple comparison test (**, *p* < 0.01; ****, *p* < 0.0001). (*N* = 8 WT, 10 KO). Note: Some animals were excluded from photometry due to inadequate fiber placement or insufficient signal. Illustrations used in this image were adapted from SciDraw.

To further investigate dopamine release during behavior, we tested mice in a fixed interval (FI) operant task, which requires waiting a set duration before a lever press will trigger reward delivery (Fig. 4a). Unlike CRF, where each lever press immediately and predictably delivers reward, the FI task dissociates the cue from reward delivery by varying the interval between them, making it a more sensitive assay for detecting how dopamine signals scale with changing cue-reward contingencies. Mice were tested across five fixed intervals (2, 4, 8, 12, and 24 s), with three sessions per interval. During each trial, the lever was extended, but only the first press after the designated interval resulted in a reward.

Both genotypes increased lever pressing as the interval lengthened and exhibited the expected “scalloping” pattern within sessions, where response rates gradually increased as the time for the next reward approached (Fig. S8). This pattern, along with the similar total lever press counts between groups (Fig. 4b), suggests that temporal processing of the interval structure was largely intact in Adgrl3 KO mice.

Significant genotype differences emerged in latency measures. *Adgrl3* KO mice took longer to initiate lever pressing across intervals (Fig. 4c) and showed increased average press latency across sessions (Fig. 4d). Both groups, however, displayed similar patterns across intervals, indicating that these differences are not due to impaired adaptation to the changing contingencies. KO mice also exhibited longer reward-retrieval latencies (Fig. 4e). These results suggest reduced task engagement or delayed response execution.

We next examined dopamine release during the FI task using dLight1.2 fiber photometry. When aligned to lever extension, dopamine signals decreased with increasing interval length, consistent with reduced reward certainty. WT mice showed higher dopamine levels at shorter intervals and a larger drop across intervals (ΔAUC = 68 units), whereas KO mice started lower and showed a smaller decrease (ΔAUC = 39 units) (Fig. 4f, g). We next aligned the signal traces to dipper extension, defined as the moment of reward delivery. Both genotypes showed increasing dopamine responses as the fixed interval and the delays between cue and reward lengthened. However, WT mice showed a larger increase (ΔAUC = 82 units) compared to *Adgrl3* KO mice (ΔAUC = 14.5 units) (Fig. 4i–k). Together, these findings show that *Adgrl3* KO mice fail to scale cue-evoked dopamine signals to task contingencies, despite intact interval-dependent changes in press latencies (Fig. 4c, d).

### Amphetamine challenge suggests comparable dopamine release capacity in WT and *Adgrl3* KO mice

**Fig. 5:**
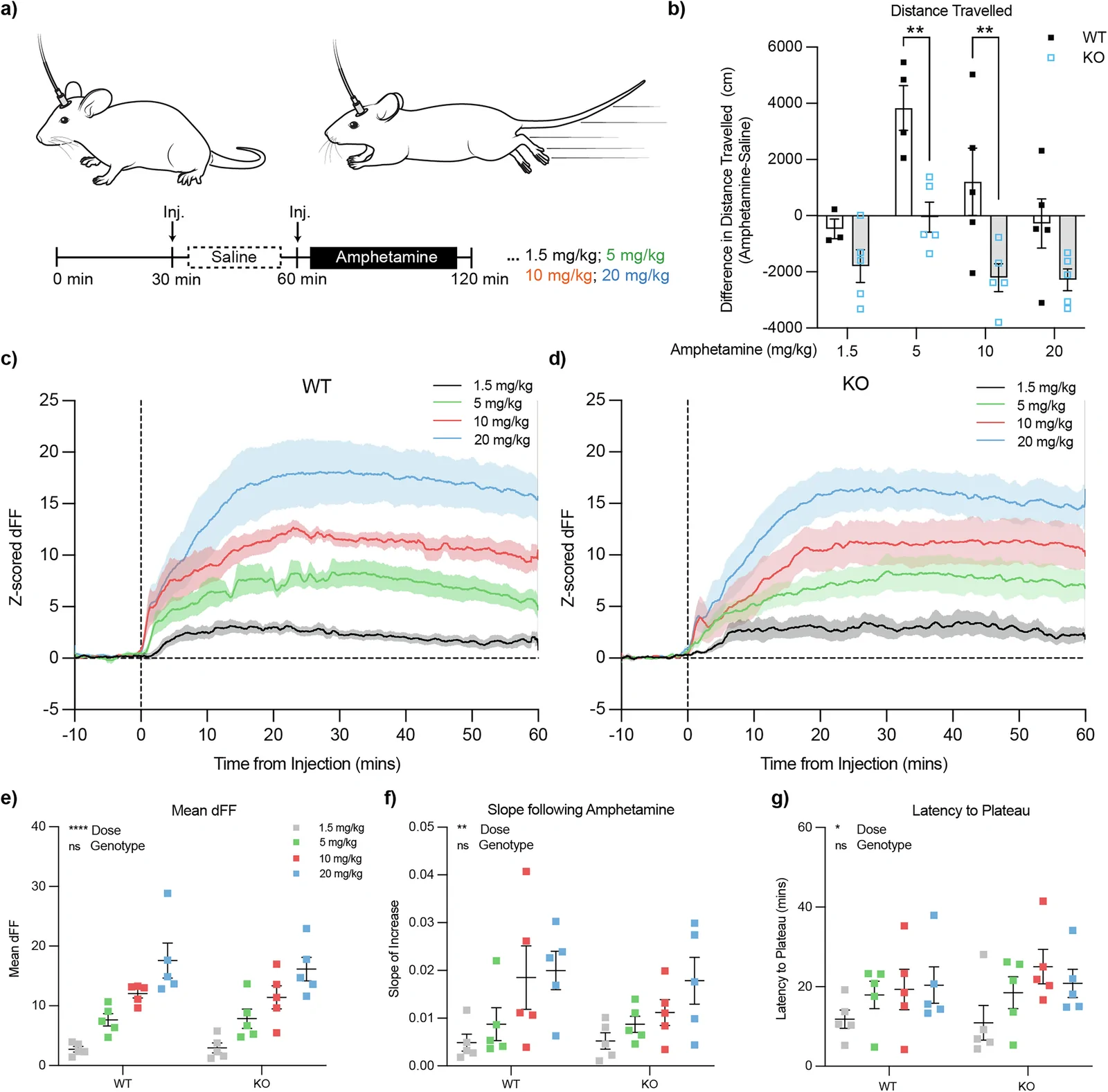
Open field test with amphetamine challenge suggests phasic dopamine release capacity is similar between WT and *Adgrl3* KO mice. **a** Mice injected with AAV5-hSyn-dLight1.2 and implanted with an optic fiber in the nucleus accumbens were used to measure dopamine responses to amphetamine in an open field test. Following a 30-min habituation period, mice received a saline injection to assess the effects of injection stress on dopamine signaling, followed by amphetamine administration (1.5, 5, 10, or 20 mg/kg subcutaneous) on separate days. Dopamine activity was recorded using fiber photometry, and locomotor behavior was tracked with AnyMaze software (Stoelting Co., Wood Dale, IL). **b** Difference in average distance traveled by WT and *Adgrl3* KO mice injected with saline versus varying doses of amphetamine. Statistical analysis was performed using a two-way ANOVA followed by Ṥídák’s multiple comparison test (**, *p* < 0.01). ****, *p* < 0.0001 for genotype and ***, *p* < 0.001 for dose. (*N* = 5 WT, 5 KO). Some data points were excluded due to inadequate video analysis. Average dLight1.2 traces aligned to amphetamine injection for **c** WT and **d** *Adgrl3* KO mice. **e** Mean dF/F, **f** slope, and **g** latency to reach plateau for WT and *Adgrl3* KO mice following amphetamine injection at the four doses. Statistical analysis was performed using a two-way ANOVA (*, *p* < 0.05; **, *p* < 0.01; ****, *p* < 0.0001). (*N* = 5 WT, 5 KO). Illustrations used in this image were adapted from SciDraw.

Our ex vivo FSCV findings indicated increased dopamine release capacity in KO mice (Fig. 2), yet in vivo photometry revealed reduced cue-evoked dopamine. To determine whether release capacity is altered in vivo, we administered increasing doses of amphetamine (1.5, 5, 10, and 20 mg/kg, s.c.) and monitored dopamine dynamics using fiber photometry (Fig. 5). Locomotor activity was simultaneously tracked in an open field using AnyMaze.

In WT mice, 5 mg/kg and 10 mg/kg doses increased locomotion, while 20 mg/kg reduced activity, consistent with well-established stereotypy observed at high doses [30]. In contrast, *Adgrl3* KO mice showed reduced locomotor activity across all doses (Fig. 5b), suggesting altered behavioral sensitivity to amphetamine.

Despite these behavioral differences, dopamine signal traces aligned to amphetamine injection revealed no significant genotype differences in mean dFF, slope, or latency to plateau (Fig. 5c, d). These data indicate that dopamine release capacity is comparable between genotypes and rule out sensor saturation as an explanation for the reduced dopamine signals observed during the FI task.

We also analyzed spontaneous dopamine release (Fig. S9). The total number of spontaneous events did not differ significantly between WT and *Adgrl3* KO mice. Event frequency decreased with amphetamine in both genotypes, especially at higher doses. Event duration increased dose-dependently, but peak amplitude remained unchanged.

## Discussion

Published work using *Adgrl3* knockout models is generally interpreted as supporting a hyperdopaminergic phenotype. While several aspects of our data are consistent with this view, we also observed reduced dopamine signaling under specific experimental conditions. These findings indicate that ADGRL3 shapes striatal dopamine dynamics in a context-dependent manner.

### Methodological considerations in interpreting divergent dopamine signals

An important framework for interpreting these results comes from methodological distinctions between FSCV and fiber photometry. Recent work directly compared FSCV to dLight-based photometry in dorsal striatal slices and reported similar input-output relationships for electrically evoked dopamine release under baseline conditions [31]. However, pharmacological inhibition of the dopamine transporter (DAT) revealed important differences: FSCV detected increased peak dopamine levels, whereas photometry reported prolonged signal decay without changes in peak amplitude.

This divergence was attributed to increased dopamine diffusion and effective sampling volume at the FSCV electrode. Accordingly, the authors concluded that FSCV preferentially detects dopamine signals that diffuse away from release sites (non-synaptic dopamine overflow), whereas fiber photometry using genetically encoded dopamine sensors reports dopamine changes at synaptic and some extrasynaptic membranes near functional release sites [31]. These findings provide a precedent for apparent mismatches between FSCV and dLight-based fiber photometry measurements of dopamine dynamics.

In the hippocampus, ADGRL3 organizes specific excitatory synapses through trans-synaptic interactions with teneurins and FLRTs [4]. Presynaptic teneurins promote recruitment of the active-zone scaffolding protein RIM1[9], thereby linking the trans-synaptic adhesion complex to synaptic release machinery. If a similar mechanism operates at dopaminergic terminals, where RIM and related active-zone proteins scaffold release sites [32], loss of ADGRL3 could disrupt the formation of synapse-like terminal structures and allow dopamine to disperse into a larger extracellular volume. Although DAT remains expressed on these disorganized terminals [33], the dispersed dopamine would expand the effective sampling volume of the carbon-fiber electrode, resulting in a larger FSCV peak amplitude through the mechanism Salinas et al. proposed for pharmacological DAT blockade. Conversely, less dopamine would reach dLight1.2 at synaptic membranes, consistent with the blunted cue-evoked dopamine signals we observed in vivo.

Importantly, the upstream causes differ between these scenarios. Pharmacological DAT blockade globally reduces transporter activity, slowing both per-event and bulk dopamine clearance and increasing both peak amplitude and duration. By contrast, in *Adgrl3* KO mice under the proposed mechanism, DAT density and activity remain intact. While altered terminal organization could increase peak amplitude, overall DAT capacity per unit tissue would be unchanged, preserving bulk clearance. This may explain the unchanged transient duration in slice recordings and the normal amphetamine-evoked response in vivo. Direct analysis of dopaminergic synaptic architecture in *Adgrl3* KO mice will be necessary to test this hypothesis. More broadly, these findings reframe ADGRL3 as a candidate structural organizer of dopaminergic terminals, extending its established role at synapses.

Beyond sensor-specific differences, acute slice preparations lack physiological tonic and phasic firing patterns of dopamine neurons and rely on electrical stimulation that activates not only dopaminergic terminals but also cholinergic and other neuromodulatory inputs that directly regulate dopamine release [34, 35]. Such conditions may exaggerate dopamine output relative to the intact circuitry present in vivo. Moreover, neuromodulatory processes involving nicotinic acetylcholine receptor desensitization [36, 37], D2 autoreceptor feedback [38–40], or calcium dynamics, may be altered in *Adgrl3* KO mice and differentially engaged in slices versus behaving animals. In contrast, in vivo recordings preserve ongoing synaptic input and feedback loops that shape dopamine dynamics during behavior and may outweigh terminal-level effects.

### Preserved dopamine release capacity and reuptake

Although we observed alterations in dopamine release using both ex vivo and in vivo approaches, we did not observe changes in dopamine reuptake. Moreover, dopamine release capacity, assessed using an amphetamine challenge with concurrent in vivo photometry, was preserved in *Adgrl3* KO mice. Together, these findings are consistent with intact terminal release mechanisms and argue against reduced dopamine availability as an explanation for blunted cue-evoked dopamine signals observed during behavior. Future studies could further validate this conclusion using orthogonal approaches, such as fluorescent false neurotransmitters to quantify dopamine release at individual synapses [41].

The absence of reuptake differences was somewhat unexpected, given prior work in an *Adgrl3* KO rat model in which FSCV measurements of spontaneous dopamine release showed faster reuptake, attributed to increased striatal dopamine transporter expression [17, 18]. Several methodological differences may account for this discrepancy. Spontaneous dopamine fluctuations in slices occur at lower concentrations and over longer durations [42, 43], allowing dopamine transporters to operate below saturation and making differences in reuptake kinetics more detectable. In contrast, electrically evoked release produces brief, high-concentration dopamine release that can saturate the transporter, masking subtle kinetic changes.

Notably, we also observed no genotype-dependent differences in spontaneous dopamine events in vivo, where transporter saturation is unlikely. Consistent with this, a recent amperometric study in anesthetized *Adgrl3* KO rats reported no differences in reuptake kinetics in the nucleus accumbens [44]. Taken together, these findings suggest that ADGRL3 does not substantially alter dopamine clearance under the conditions tested.

### Additional considerations and limitations

Several additional factors should be considered when interpreting the present findings. First, ADGRL3 expression is highest during early development [2], yet the present study examined the effects of *Adgrl3* loss on dopamine signaling primarily in adulthood. Because prior studies of ADGRL3 function tested different developmental windows, direct comparison of reported phenotypes across studies is challenging. A systematic assessment of dopamine signaling across development, together with temporally controlled genetic approaches such as an inducible adult *Adgrl3* knockout, will therefore be required to determine whether the dopamine phenotypes observed in *Adgrl3* KO mice reflect early developmental effects or arise from ADGRL3 function later in life.

Second, our work primarily focused on the nucleus accumbens, which receives dopaminergic input from the ventral tegmental area. While similar mechanisms may apply to the dorsal striatum, this region is innervated predominantly by dopamine neurons from the substantia nigra pars compacta and contributes to distinct behavioral domains. As such, the extent to which the present findings generalize across striatal subregions remains to be determined.

Finally, we note that our dopamine sensor was expressed broadly, and thus the current study cannot distinguish how ADGRL3 may differentially influence dopamine signaling across discrete striatal cell populations. Future studies using cell-type-specific genetic or pharmacological approaches will be required to define how ADGRL3 functions within discrete striatal pathways. In addition, while viral expression may have extended beyond the nucleus accumbens proper, dopamine signals were sampled optically at fiber locations targeted to the nucleus accumbens; nevertheless, contributions from adjacent striatal regions cannot be entirely excluded.

### Effects of *Adgrl3* loss on dopamine-related behaviors

*Adgrl3* KO mice have exhibited disruptions across multiple dopamine-modulated behaviors, including motor control, learning, and motivation [14–16]. We replicated some of these phenotypes, most notably increased activity, a hallmark of the *Adgrl3* KO model. We also found that *Adgrl3* KO mice weighed significantly less than WT controls (Fig. 1a).

We used PiezoSleep monitoring to show that *Adgrl3* KO mice exhibit increased activity during the dark phase, and increased sleep with longer sleep bouts during the light phase, consistent with altered light-entrained sleep-wake patterns. This pattern may reflect increased sensitivity to light or compensatory rest following elevated activity during the dark phase. Given dopamine’s role in sleep-wake regulation, future studies using constant-darkness conditions will be important to determine whether free-running circadian rhythms are altered in *Adgrl3* KO mice.

We next examined goal-directed behavior. *Adgrl3* KO mice had higher CRF response rates that normalized with training (Fig. 3b, c), and on the fixed-interval task, they showed normal lever-press counts but longer latencies to initiate lever press and retrieve reward (Fig. 4c–e). Lever and dipper extensions act as discrete cues guiding behavioral responses. Reduced dopamine release at lever and dipper extension observed in *Adgrl3* KO mice may underlie their longer response latencies (Fig. 4f, g, i, j), consistent with the inverse relationship between lever-press latency and dopamine transient magnitude observed during the CRF task (Fig. S7). The delay may also reflect a more generalized deficit in cue-guided response. Whether this arises from altered motivation, attentional deficits, or changes in temporal processing cannot be distinguished with the current data. Notably, similar impairments are reminiscent of task-switching difficulties reported in individuals with ADHD, where inattention contributes to challenges in smoothly transitioning between actions [45].

Surprisingly, when challenged with amphetamine, *Adgrl3* KO mice showed a suppression in activity compared to WT controls despite comparable changes in dopamine levels (Fig. 5b). This finding aligns with previous reports in *Adgrl3* KO rats, which also showed blunted locomotor response to amphetamine [17]. Because amphetamine drives efflux directly through reverse transport and amphetamine-evoked dopamine release was comparable between genotypes (Fig. 5c, d), the altered locomotor response in KO mice likely reflects differences downstream of dopamine release. Notably, amphetamine treatment in individuals with ADHD also reduces hyperactivity [46, 47].

Finally, we encountered significant challenges in maintaining a colony of *Adgrl3* KO mice. Although previous studies have not reported breeding difficulties, we were unable to obtain viable litters from homozygous KO breeding pairs. Female *Adgrl3* KO mice consistently showed poor maternal behavior, often displaying agitation when housed with males and, in many cases, cannibalizing their pups shortly after birth. As a result, we maintained the colony using heterozygous breeding pairs or by pairing a homozygous KO male with a heterozygous female, which still yielded limited success. While maternal behavior is not strictly a result of intact dopamine signaling, the mesolimbic dopamine system is known to play a role in maternal motivation [48]. Future studies should consider using region- or cell-type-specific *Adgrl3* knockouts to improve breeding viability and refine mechanistic interpretations.

## Supplementary information

Supplemental Material

## Data Availability

All data needed to evaluate the conclusions in the paper are present in the paper and/or the Supplementary Materials. Data will be shared upon request. Contact the corresponding authors here: jaj2@cumc.columbia.edu, ck491@cumc.columbia.edu.
