# Supplementary material for: Altered striatal dopamine regulation in Adgrl3 knockout mice
Source: Neuropsychopharmacology. 2026 Jun 26;51(11):2248–57. doi: 10.1038/s41386-026-02474-3 (PMC13598071; doi:10.1038/s41386-026-02474-3)
Supplement: Supplementary file 1 [file 41386_2026_2474_MOESM1_ESM.pdf]

Supplementary Materials for

Altered striatal dopamine regulation in *Adgrl3* knockout mice

Nicole Perry-Hauser *et al.*

*Corresponding authors, e-mail: jaj2@cumc.columbia.edu, ck491@cumc.columbia.edu

This PDF file includes:

Supplementary Materials and Methods
Figs. S1 to S9

Supplementary Materials and Methods

Open Field. Locomotor activity (6-12 weeks) was assessed for 60 mins in a 42×42×38 cm plexiglass enclosure equipped with infrared photobeams (Med Associates). Activity was recorded using Motor Monitor software (Kinder Scientific). Horizontal movement, rearing, and center (21×21 cm) vs. periphery time were quantified over the full session.

PiezoSleep Study. Sleep/wake cycles were recorded over five consecutive days using the PiezoSleep system (Signal Solutions LLC) following established protocols [1,2]. Mice were singly housed under a 12-hour light/dark cycle (6:00-18:00). Floor sensors recorded sleep bouts, respiration, temperature, and movement. Activity counts reflected movement intensity per 10-minute bin, with values between 0.0 and 0.3 representing the range of signal amplitudes detected by the PiezoSleep sensor. Interdaily stability and intradaily variability were calculated as previously described [3].

Gustometer. Mice were mildly water-restricted using 2% w/v citric acid water the day before training. Mice were weighed daily and monitored for normal behavior. On Day 1, 2–3 mice were trained in the "Davis Rig" gustometer (Med-Associates) for 30 minutes to learn licking behavior, with sipper access for 5–15 seconds per trial followed by a fixed intertrial interval. On Day 2, individual training with two sippers followed the same protocol. On testing day, mice completed a 6-bottle choice task with evaporated milk concentrations (0-100%) presented in pseudorandom order over 30 minutes, with 20-second intertrial intervals.

Fast-scan Cyclic Voltammetry (FSCV). Male and female mice (3-6 months) were euthanized by cervical dislocation. Brains were rapidly extracted and sectioned (250 µm coronal slices including striatum) in ice-cold oxygenated sucrose solution (10 mM NaCl, 2.5 mM KCl, 25 mM $NaHCO_3$, 0.5 mM $CaCl_2$, 7 mM $MgCl_2 \cdot 6H_2O$, 1.25 mM $NaH_2PO_4 \cdot 6H_2O$, 180 mM sucrose, and 10 mM glucose), which was oxygenated with a 95% O_2/5% CO_2 mixture and adjusted to a pH of 7.4. Each brain section was allowed to recover for 30 minutes at 34°C in artificial cerebrospinal fluid (ACSF; 125 mM NaCl, 2.5 mM KCl, 25 mM $NaHCO_3$, 1.5 mM $CaCl_2$, 1 mM $MgCl_2 \cdot 6H_2O$, 1.25 mM $NaH_2PO_4 \cdot 6H_2O$, and 10 mM glucose). Slices were then bubbled in oxygenated ACSF at room temperature for at least 30 min prior to transfer to a recording chamber.

FSCV recordings followed established protocols [4,5]. Slices were perfused with ACSF at 2 mL/min at 34°C. A bipolar concentric electrode (Pt/Ir; 400 µm OD) was placed in dorsal or ventral striatum, and a carbon fiber electrode (5 µm diameter, 100-200 µm length) was positioned ~150 µm away, 50 µm deep. A triangular waveform (-450 mV to +800 mV over 8.5 ms, 294 V/s) was applied every 100 ms. Signals were amplified (Axopatch 200B), filtered (10 kHz Bessel), digitized (25 kHz, ITC-18), and analyzed using custom Igor Pro scripts (WaveMetrics Inc).

Stimulation was delivered via Iso-Flex stimulus isolator (AMPI) triggered by TTL pulses. Single pulses (0.1-1.0 ms, 200 µA) were delivered every 2 minutes for 10 minutes; the middle three peaks were analyzed. Electrodes were calibrated using 1 µM dopamine in 0.1% HCl.

Stereotaxic Surgery and Fiber Implantation. Mice (≥8 weeks old) were anesthetized with 3% isoflurane (500 mL/min), maintained at 1-2% (100 mL/min). Using a Hamilton syringe and nanoliter injector, 375 nL of AAV5-hSyn-dLight1.2 (Addgene #111068-AAV5) was injected unilaterally into the nucleus accumbens at AP +1.7 mm, ML ±1.2 mm, DV, -4.2, -4.1, and -4.0 mm (125 nL/site), with 1-minute spacing and a 5-minute post-injection pause.

Following injection, 400 µm fiber optics (Doric Lenses, 5.0 mm FLT tip) were implanted at -4.1 mm DV and secured with dental cement and mini screws. Mice received 500-1,000 µL saline and recovered on a heating pad for ≥15 minutes. Post-op monitoring lasted ≥3 days, with carprofen (5 mg/mL) administered as

needed. Behavioral training began ~4 weeks post-surgery. After experiments, mice were perfused with 4% paraformaldehyde, and brains were processed to verify viral expression and fiber placement.

In Vivo Fiber Photometry. Fiber photometry setup followed established protocols [6]. A 4-channel LED Driver (Doric Lenses) controlled 405 nm (isosbestic) and 465 nm (dLight1.2) LEDs via bandpass filters and fluorescent minicubes. LEDs were modulated at 210 Hz (405 nm) and 330 Hz (465 nm) using Synapse software and RZ5P processors (Tucker-Davis Technologies). Light power at the patch cord was 6-12 µW (405 nm) or 30 µW (465 nm), respectively. Emitted fluorescence passed through 500-540 nm GFP filters to photodetectors (DC low gain), digitized at 1017 Hz, and demodulated with a 6 Hz low-pass filter.

Data were analyzed using custom MATLAB scripts. Signals were downsampled 10X; the 405 nm channel corrected for motion and photobleaching. To calculate the change in fluorescence (dFF), a fitted 405 nm signal was generated using a least-squared linear regression of the 405 nm signal against the 465 nm signal. This fitted control was then used to normalize the 465 nm signal using the follow formula:

$$dFF = 100 \times (465 \text{ nm signal} - \text{fitted } 405 \text{ nm signal}) / \text{fitted } 405 \text{ nm signal}$$

Resulting dFF traces were z-scored to account for variability in expression or fiber placement.

Z-scored dFF was aligned to behavioral events (lever extension, dipper presentation, reward delivery). Baseline (-5 to -0.5 s before event) was subtracted, and traces were averaged across trials and sessions. Peak amplitude and area under the curve (AUC) were calculated within post-event windows: 0-1.5 s (lever), 0-1 s (dipper), and 0-3 s (reward).

Spontaneous events during ITIs were detected using MATLAB "findpeaks", selecting peaks >2 SD above session mean. For each, a ±2 s window was extracted, and prominence, amplitude, and width were quantified.

Operant Apparatus. Operant tasks (CRF and FI) were conducted in sound-attenuated chambers (Med-Associates) equipped with retractable levers, a liquid dipper (~15 µL evaporated milk), and an LED house light. Levers were positioned 5 cm from the feeder trough; head entries were detected using an infrared sensor. Chambers were controlled and data recorded using Med-PC software.

Adult mice (>90 days) were trained to access the dipper, then to lever press under a continuous reinforcement schedule, followed by fixed interval training. Mice were weighed daily and food restricted to 80-90% of baseline weight; water remained unrestricted.

Dipper Training. Mice completed four sessions of dipper training with "free" access to evaporated milk (10 s per dipper presentation). Session 1 included 30 presentations with 30 s variable ITIs; sessions 2-4 included 60 presentations. Head entries and latencies were recorded.

Continuous Reinforcement (CRF). Mice were trained to lever press under a CRF schedule, receiving evaporated milk (5 s dipper access) after each press. Levers retracted post-reinforcement and reappeared after variable ITIs averaging 20 s. Initial sessions ended after 30 reinforcements or 30 minutes. Later sessions after 60 reinforcements or 1 hour. Training continued daily until the mice consistently earned 60 reinforcements.

Fixed Interval (FI) Task. Following CRF training, mice were tested on FI schedules (2, 4, 8, 12, 24 s), each repeated across three sessions. Mice could earn up to 60 reinforcements or complete a 1-hour session. After each reinforcement (10 s dipper access), levers retracted and reappeared after variable ITIs, averaging 12 s. Lever presses during the fixed interval were recorded; levers retracted only after the interval elapsed.

Open Field Test with Amphetamine Challenge and Fiber Photometry. A subset of mice with AAV5-hSyn-dLight1.2 injections and fiber implants in the nucleus accumbens (AP +1.7 mm, ML ±1.2 mm, DV, -4.2, -4.1, and -4.0 mm; 125 nL/site) were used to assess dopamine responses to amphetamine. Mice received subcutaneous (s.c.) injections of amphetamine (1.5, 5, 10, 20 mg/kg in 0.9% saline) on separate days, following a protocol adapted from recent comparative studies of microdialysis and photometry [7].

On each test day, mice were weighed, tethered to the fiber photometry setup, and placed in a 24×45×20 cm enclosure with ~0.5 cm bedding. After a 30-minute habituation period, video tracking (AnyMaze software) and photometry recordings were initiated. Mice were then injected with 0.9% saline (s.c.) and monitored for 30 minutes to control for injection-related stress. Finally, amphetamine was administered (s.c.), and the mice were monitored for 60 minutes.

Histology. Adult mice were anesthetized with ketamine (100 mg/kg) and xylazine (10 mg/kg, i.p.), then perfused with 4% paraformaldehyde (PFA) in PBS. Brains were post-fixed overnight in 4% PFA, then stored in 0.1% sodium azide in PBS. Coronal sections (50 μm) were cut using a vibratome (Leica) at 1.0 mm amplitude and 1.0 mm/s speed.

Slices were stained overnight at 4°C with chicken anti-GFP (Abcam ab13970, 1:1,000) in blocking buffer (5% donkey serum, 0.5% bovine serum albumin, 0.1% PBS-Triton). Secondary staining used Alex Fluor 488 goat anti-chicken IgY (Thermofisher, A11039, 1:500) and DAPI (Thermofisher D1306, 1:1,000) for 1 hour at room temperature in the dark. Slices were rinsed in 50 mM Tris (pH 7.5) and mounted with ProLong Glass Antifade (ThermoFisher). Viral expression and fiber placement were confirmed via GFP staining using an SP8 confocal microscope (Leica).

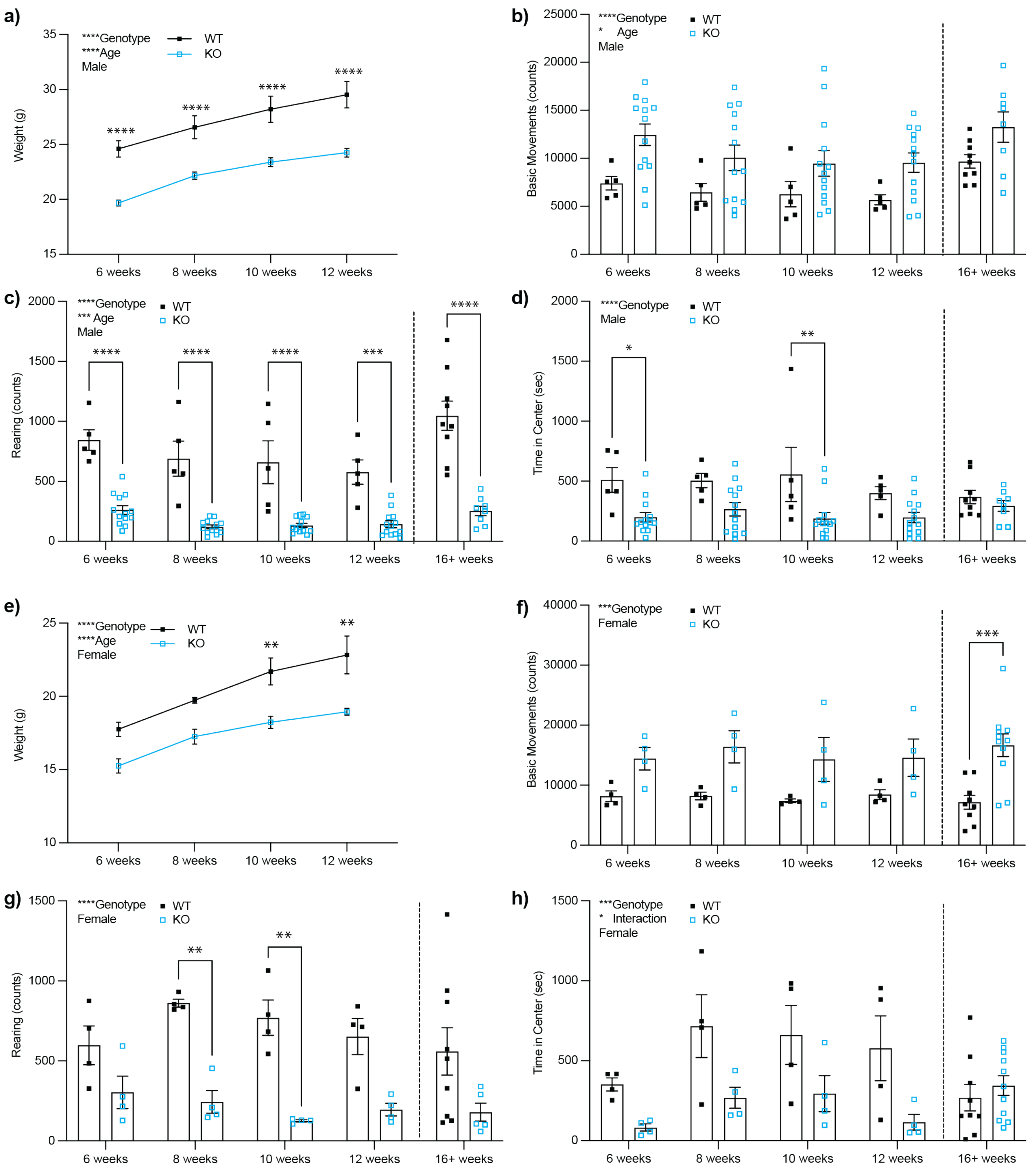

Fig. S1. ***Adgrl3*** **knockout mice show increased horizontal locomotion and decreased rearing activity across development. (a)** Weight of wild-type and *Adgrl3* knockout male mice (N=5 male WT, 13 male KO at 6-12 weeks of age; N=9 male WT, 8 male KO at 16+ weeks of age). **(b)** Horizontal locomotor activity, **(c)** rearing activity, and **(d)** time in center for WT and *Adgrl3* KO male mice across development. **(e)** Weight of wild-type and *Adgrl3* knockout female mice (N=4 female WT, 4 female KO at 6-12 weeks of age; N=10 female WT, 11 female KO at 16+ weeks of age). **(f)** Horizontal locomotor activity, **(g)** rearing activity, and **(h)** time in center for WT and *Adgrl3* KO female mice across development. A two-way repeated measures ANOVA was

performed to analyze the effect of age and genotype on each dependent variable (weight, distance traveled, rearing, or time in center). This was followed by Śídák's multiple comparisons test (*, $p < 0.05$; **, $p<0.01$; ***, $p<0.001$; ****, $p < 0.0001$).

Male and female data are combined in the main figures; sex-stratified analyses showed similar trends.

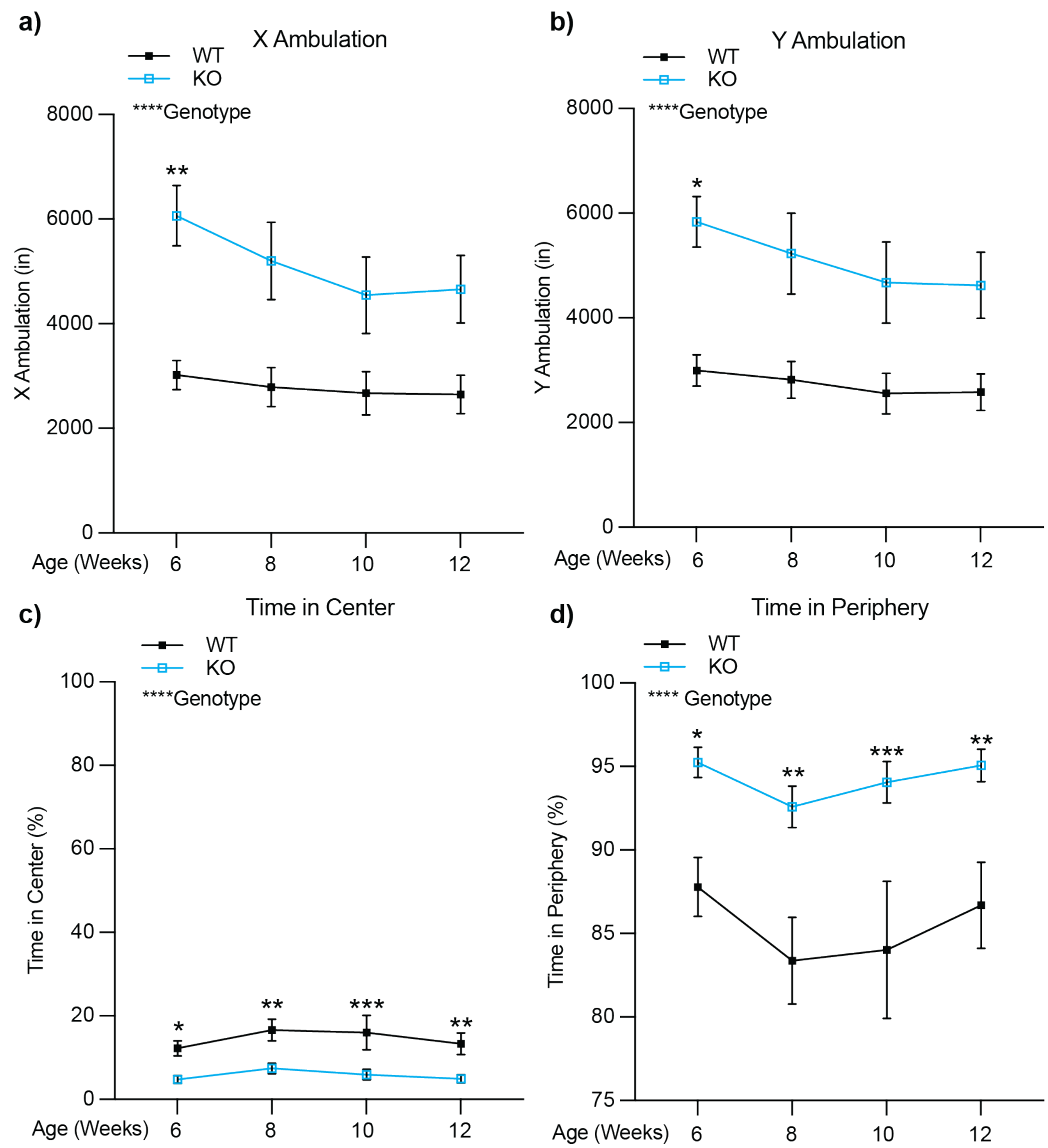

Fig. S2. *Adgrl3* knockout mice show increased X-Y ambulation and decreased time in center across development. Wild-type and *Adgrl3* knockout mice (WT: n=9; 5 males, 4 females; KO: n=17, 13 males, 4 females) **(a)** X ambulation (in inches) **(b)** Y ambulation (in inches) **(c)** time in center (%), and **(d)** time in periphery (%) across development. A two-way repeated measures ANOVA was performed to analyze the effect of age and genotype on each dependent variable (distance or percentage of time). This was followed by Śídák's multiple comparisons test (*, $p < 0.05$; **, $p<0.01$; ***, $p<0.001$; ****, $p < 0.0001$).

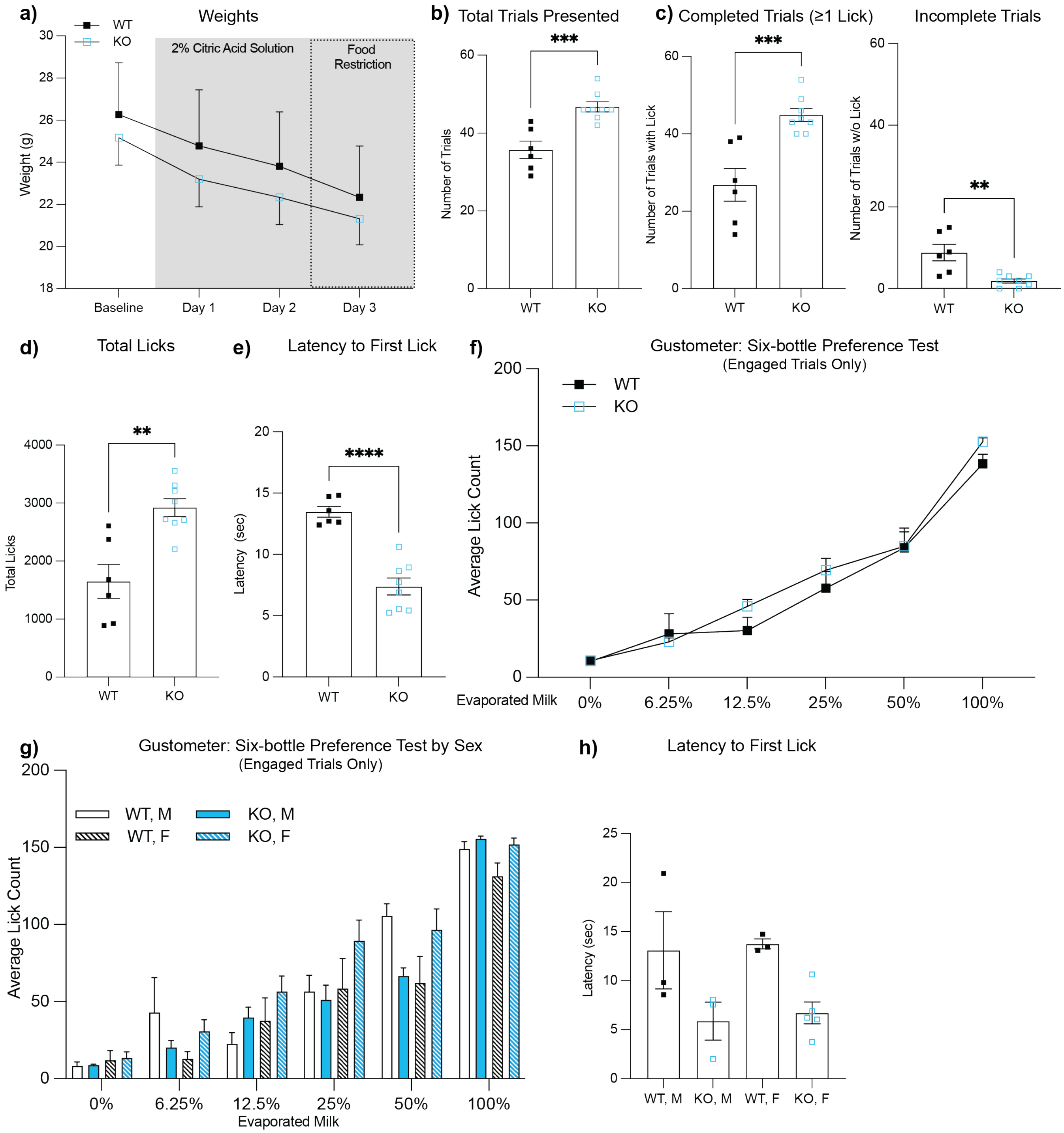

Fig. S3. *Adgrl3* knockout mice show increased lick responses but similar taste preference for an evaporated milk reward compared to WT mice. Taste preference for and licking behavior toward an evaporated milk reward were measured for WT and *Adgrl3* knockout (KO) mice using a "Davis Rig" gustometer (WT: n=6, 3 males, 3 females; KO: n=8, 3 males, 5 females). **(a)** Average weights for each genotype over the course of the study. **(b)** Number of trials presented, **(c)** number of completed and incomplete trials, **(d)** and total number of licks across the 30 min session. Statistical analysis consisted of an unpaired t test (***, p<0.001; **, p<0.01). **(e)** Average latency until first lick by genotype. Statistical analysis consisted of an

unpaired t test (****, $p<0.0001$). **(f)** Average licks by each genotype for 6 different concentrations of evaporated milk (0%, 6.25%, 12.5%, 25%, 50%, and 100%). Statistical analysis consisted of a Two-way ANOVA followed by Śídák's multiple comparison test (****, $p<0.0001$ for % of evaporated milk). No statistical difference was detected for genotype or reward. **(g)** Average licks divided by genotype and sex for 6 different concentrations of evaporated milk (0%, 6.25%, 12.5%, 25%, 50%, and 100%). Statistical analysis consisted of a Three-way ANOVA (****, $p<0.0001$ for % of evaporated milk). No statistical difference was detected for genotype or sex (N=3 WT female, 3 WT male; 5 KO female, 3 KO male). **(h)** Average latency until first lick by sex. Statistical analysis consisted of an ordinary one-way ANOVA (*, $p<0.05$).

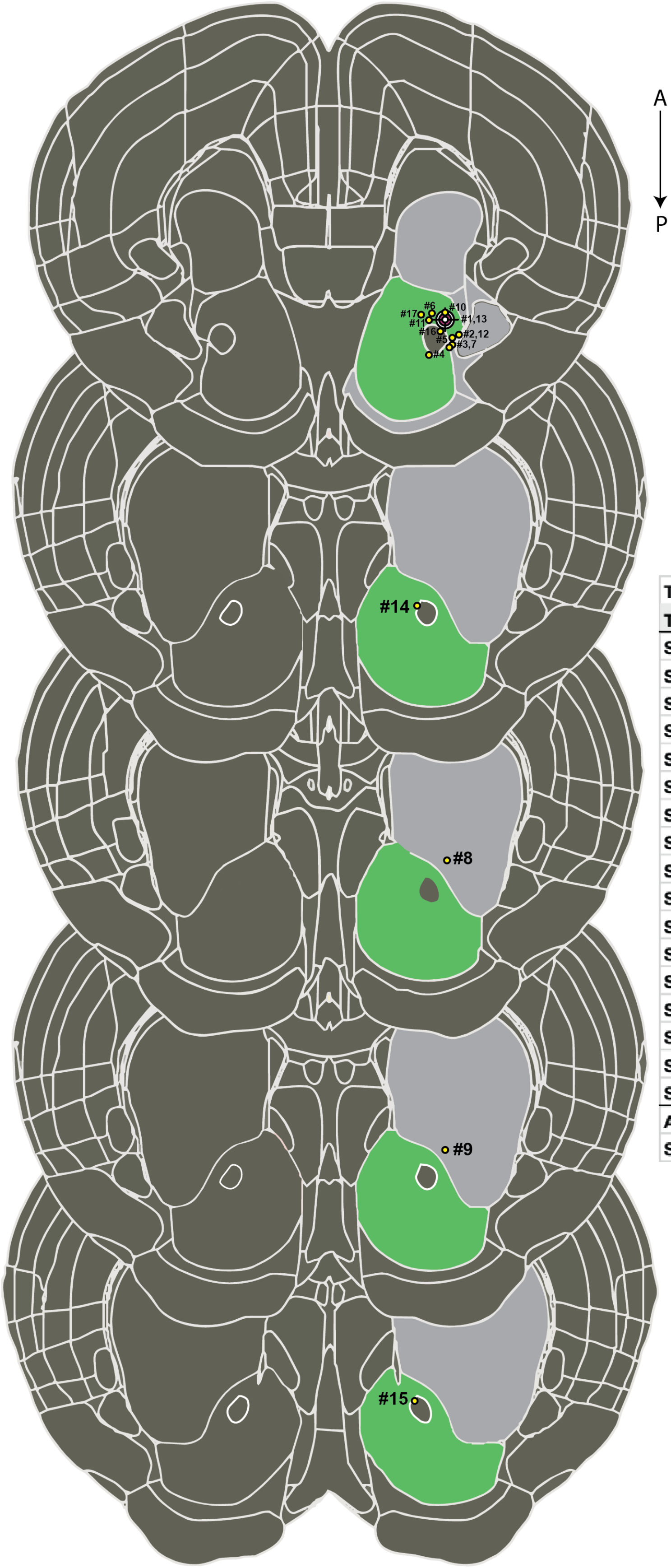

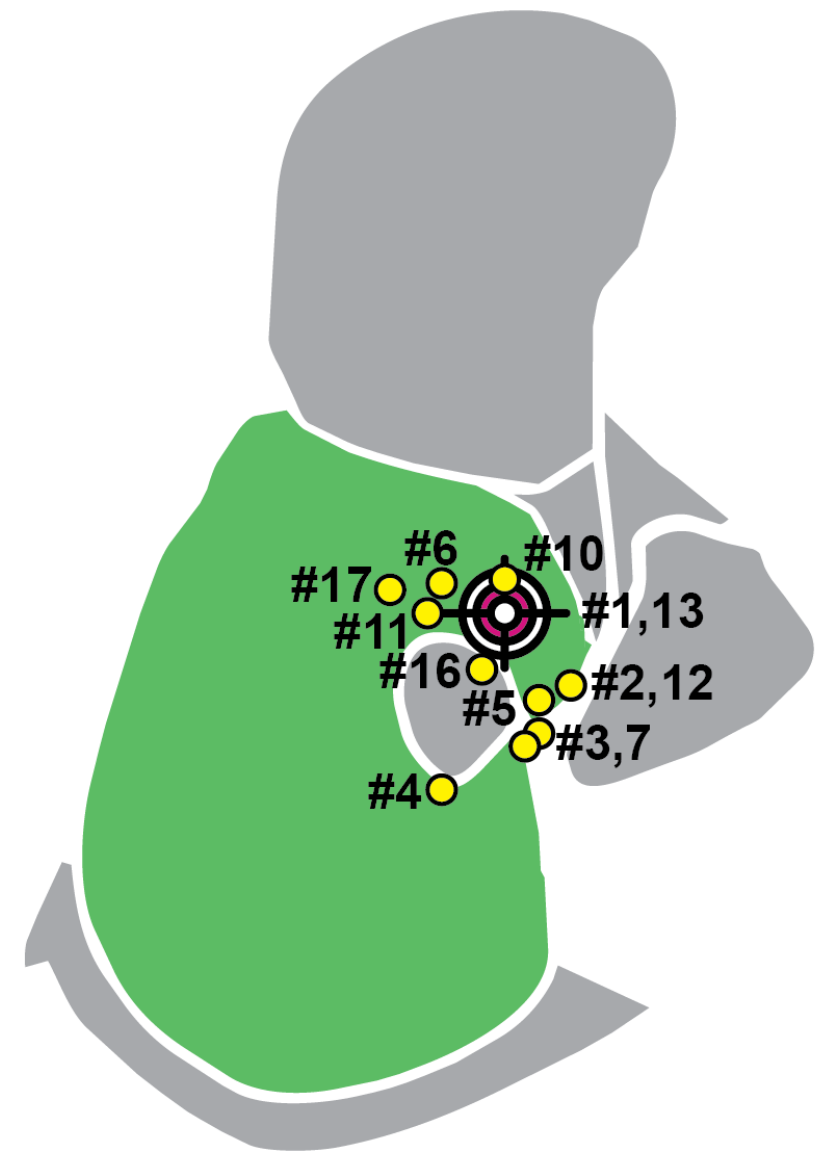

Target Name	AP (mm)	ML (mm)	DV (mm)	Region
**Target 1**	**1.7**	**1.2**	**-4.1**	**NAc**
**Sample 1**	1.70	1.20	-4.10	NAc
**Sample 2**	1.70	1.40	-4.30	NAc
**Sample 3**	1.70	1.30	-4.50	NAc
**Sample 4**	1.70	1.00	-4.50	NAc
**Sample 5**	1.70	1.30	-4.30	NAc
**Sample 6**	1.70	1.00	-4.00	NAc
**Sample 7**	1.70	1.20	-4.50	NAc
**Sample 8**	1.42	1.20	-3.90	CPu, NAc
**Sample 9**	1.34	1.20	-3.90	CPu, NAc
**Sample 10**	1.75	1.20	-4.00	NAc
**Sample 11**	1.70	0.90	-4.10	NAc
**Sample 12**	1.70	1.40	-4.30	NAc
**Sample 13**	1.70	1.20	-4.10	NAc
**Sample 14**	1.54	0.90	-4.20	NAc
**Sample 15**	1.10	0.80	-4.20	NAc
**Sample 16**	1.70	1.10	-4.30	NAc
**Sample 17**	1.70	0.80	-4.00	NAc
**Avg**	1.63	1.13	-4.18	
**Std. Dev**	0.17	0.19	0.19	

Fig. S4. Histological verification of fiber tip placement. Schematic representation of fiber optic tip locations for experimental mice included in photometry analyses (n=17) [8]**.** Fiber placement was verified histologically where tissue quality permitted. For a subset of animals, histological validation could not be obtained due to technical issues (e.g., tissue loss or damage during processing). These animals were nevertheless retained in analysis because implantation coordinates were highly consistent across subjects (A-P: 1.63±0.17 mm; M-L: 1.13±0.19 mm; D-V: -4.18±0.19 mm from bregma), and their dLight photometry signals were comparable to those observed in the group.

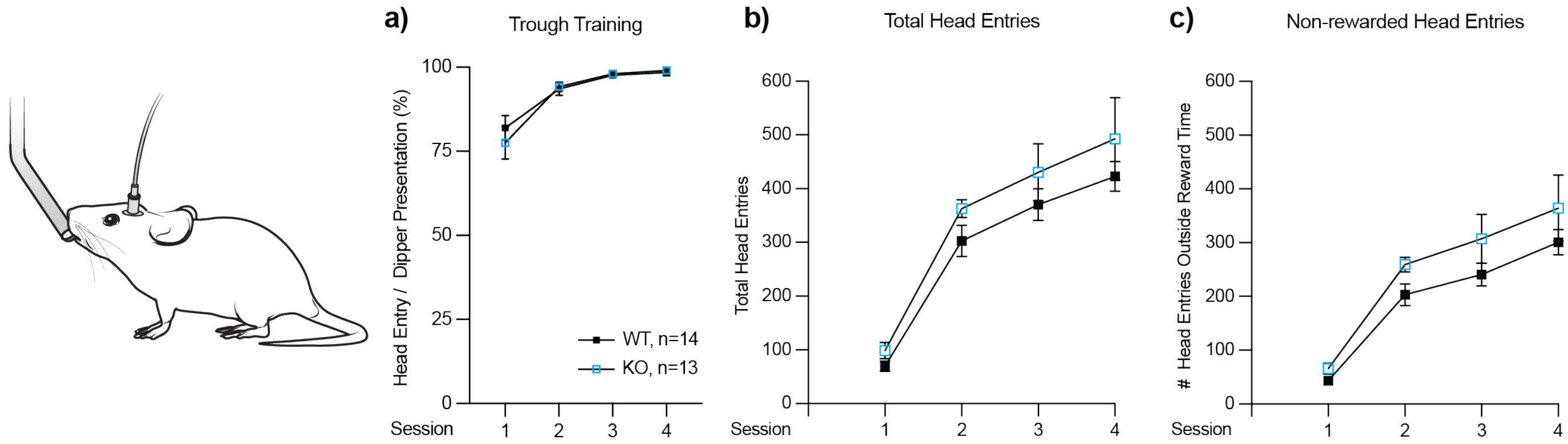

Fig. S5. *Adgrl3* knockout mice exhibit similar reward retrieval learning as WT mice. (a) Percentage of head entries relative to total possible dipper presentations. *Adgrl3* KO mice learn to retrieve an evaporated milk reward from an extended dipper at a comparable rate to WT mice. Statistical analysis was performed using a two-way ANOVA followed by Śídák's multiple comparison test (****, $p<0.0001$ for session). (N=14 WT, 13 KO). **(b)** Total number of head entries into the dipper port across sessions. **(c)** Number of head entries recorded outside of reward presentation periods.

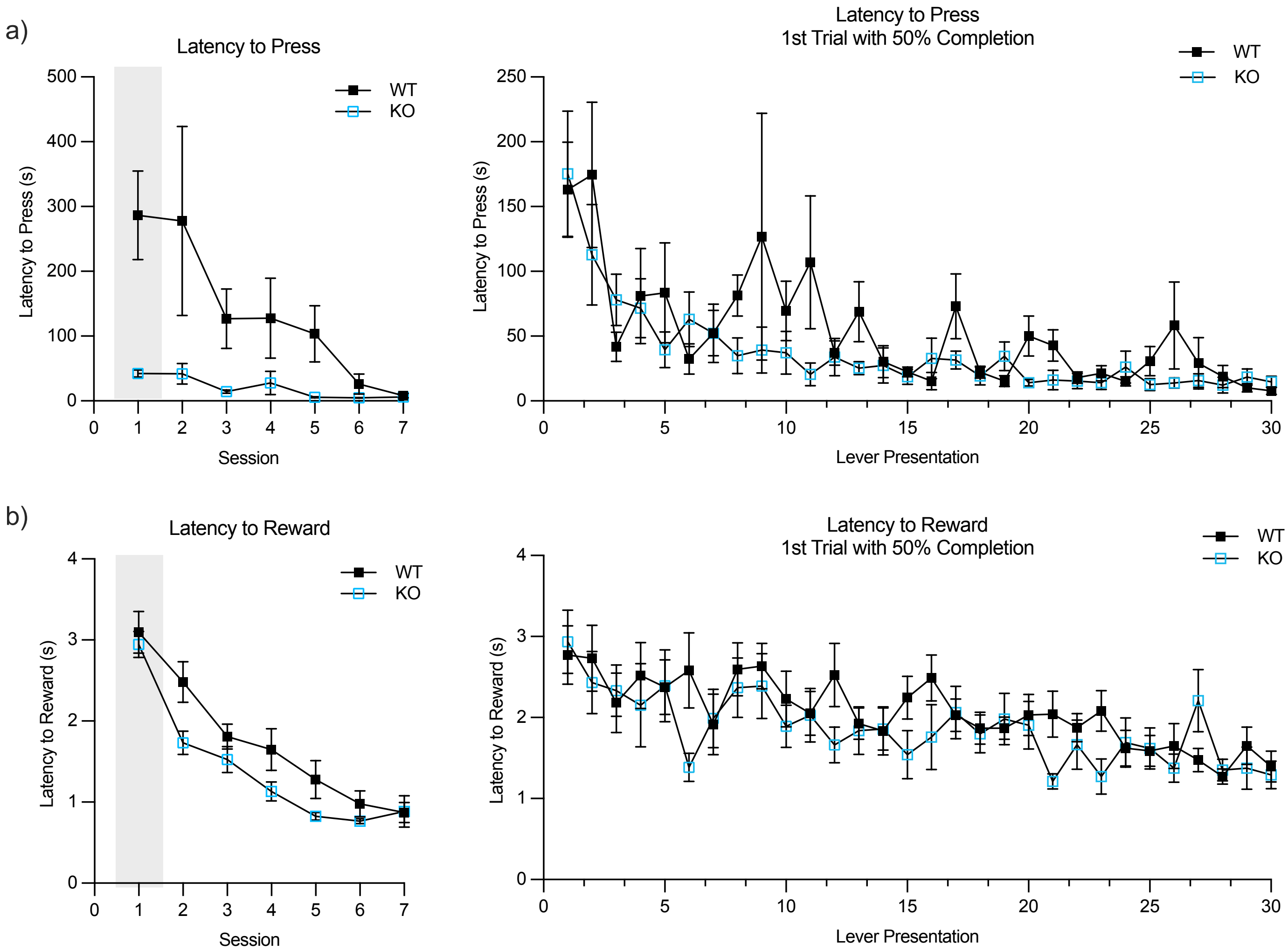

Fig. S6. Latencies to lever press and reward retrieval during continuous reinforcement training. (a) (Left) Latency to lever press (sec) across sessions for WT and *Adgrl3* KO mice. Statistical analysis was performed using a mixed effects analysis followed by Dunnett's multiple comparison test (*, $p<0.05$). (N=14 WT, 13 KO). **(Right)** Latency to lever press across trials during the first session with 50% of the trials completed. No significant difference in latency to lever press was observed under this criterion. **(b) (Left)** Latency to reward (sec) across sessions for WT and *Adgrl3* KO mice. Statistical analysis was performed using a mixed effects analysis followed by Dunnett's multiple comparison test (*, $p<0.05$; ****, p<0.00Ca01). (N=14 WT, 13 KO). **(Right)** Latency to reward across trials during the first session with 50% of the trials completed. No significant difference in reward retrieval was observed under this criterion.

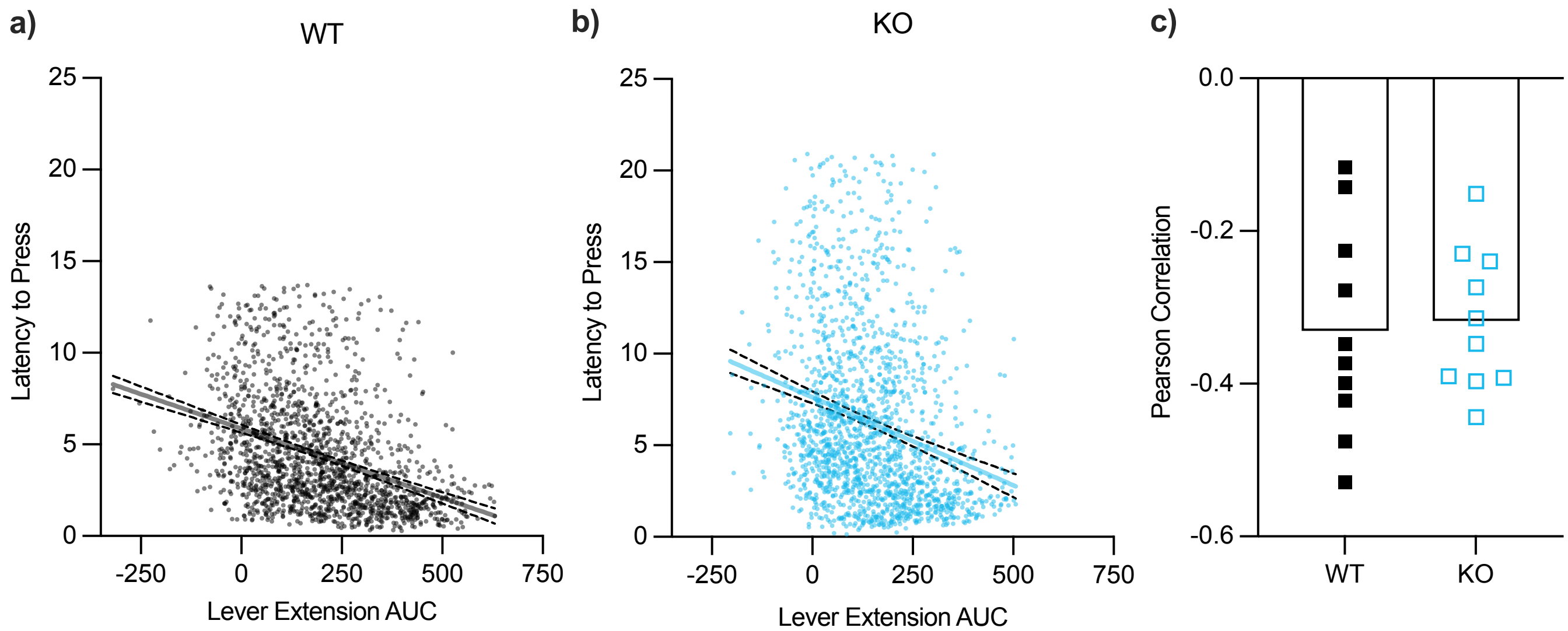

Fig. S7. Relationship between dopamine release and lever press latency during the continuous reinforcement task. Correlation between area under the curve and latency to lever press for **(a)** WT and **(b)** *Adgrl3* KO mice during the continuous reinforcement task (CRF60). Both genotypes exhibited a negative correlation, indicating that greater dopamine release was associated with reduced lever press latency. **(c)** No significant difference in Pearson correlation coefficients between WT and *Adgrl3* KO mice.

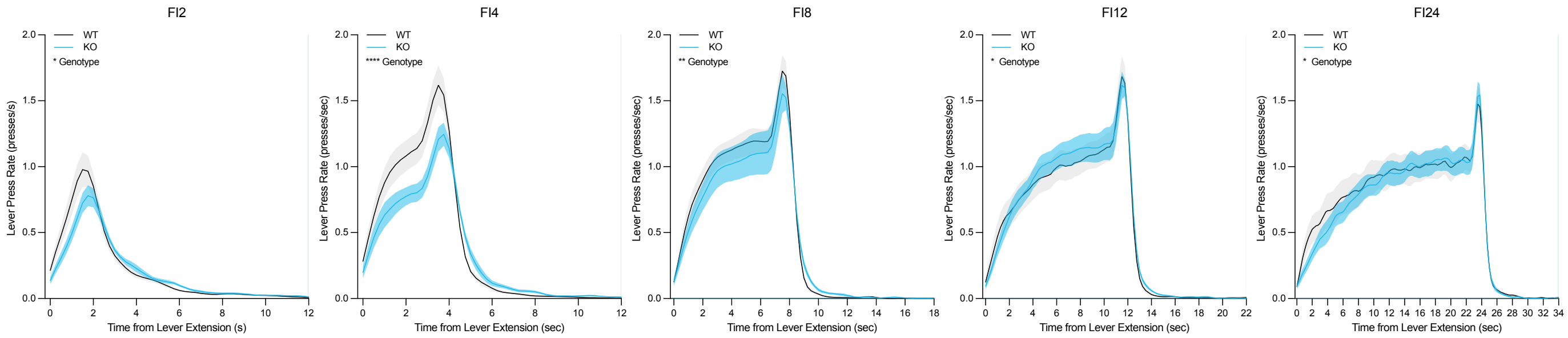

Fig. S8. Lever press rates increased as the time for the next fixed interval approached. Lever press rates (presses per second) were plotted across all fixed intervals following the initial lever extension. A characteristic "scalloping" pattern emerged, marked by an initial pause after lever extension, followed by a gradual increase in lever pressing as the fixed interval progressed. Statistical analysis was performed using a two-way ANOVA to analyze the effect of genotype and time on lever press rate (* p<0.05; **, p<0.01; ***, p<0.001; ns, not significant). (N=9 WT, 10 KO).

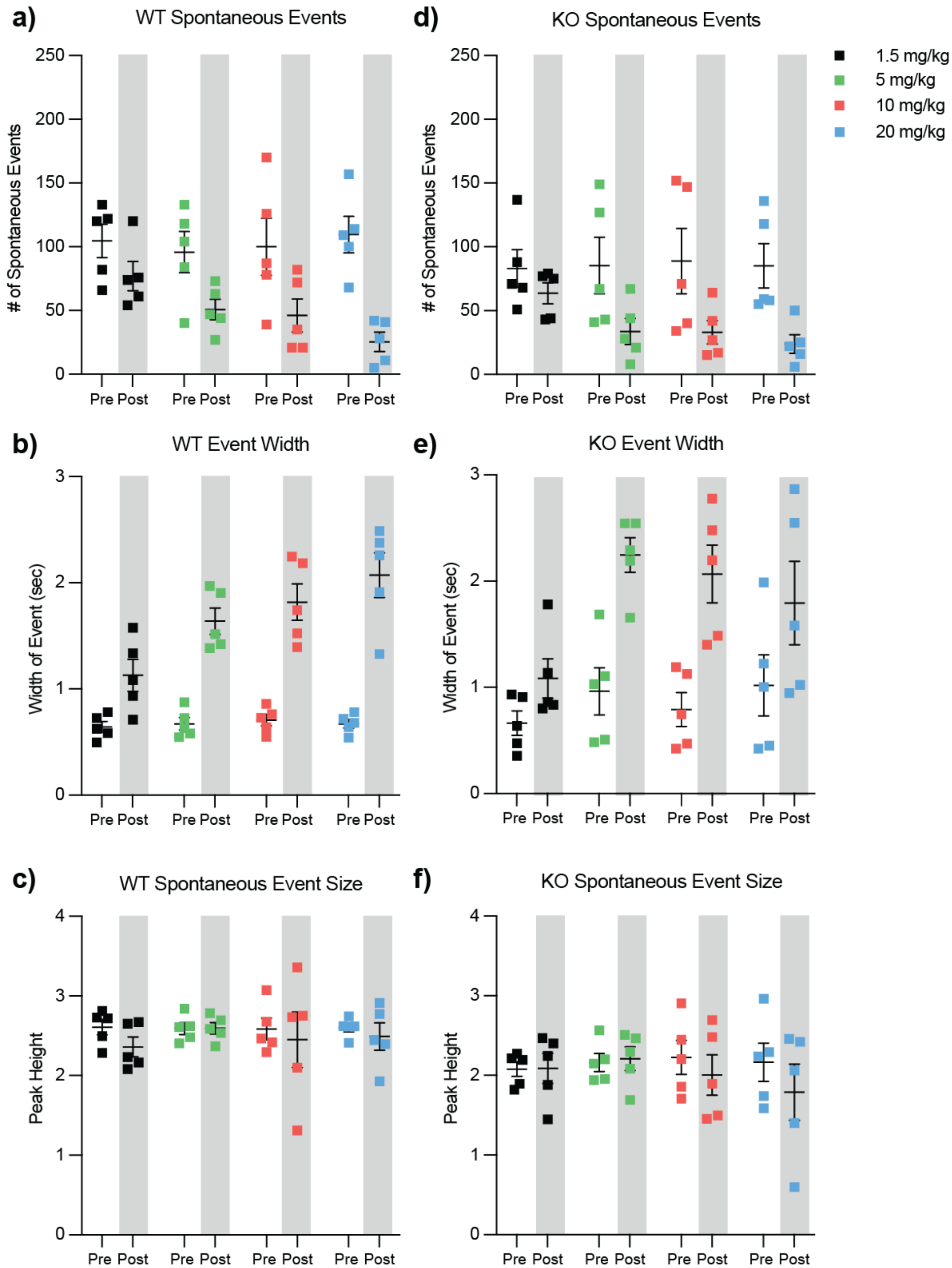

Fig. S9. Spontaneous photometry events during the open field test with amphetamine challenge showed no significant differences between WT and *Adgrl3* KO mice. (a) Total number of spontaneous events, **(b)** event duration, and **(c)** peak height for WT mice before and after amphetamine injection. **(d)** Total number of spontaneous events, **(e)** event duration, and **(f)** peak height for KO mice before and after amphetamine injection. Statistical analysis was performed using a two-way ANOVA (*, $p<0.05$; **, $p<0.01$; ****, $p<0.0001$). (N=5 WT, 5 KO).

References

1. McGowan JC, Ladner LR, Shubeck CX, Tapia J, LaGamma CT, Anqueira-González A, et al. Traumatic Brain Injury–Induced Fear Generalization in Mice Involves Hippocampal Memory Trace Dysfunction and Is Alleviated by (R,S)-Ketamine. Biol Psychiatry. 2024;95:15–26.
2. Donohue KD, Medonza DC, Crane ER, O'Hara BF. Assessment of a non-invasive high-throughput classifier for behaviours associated with sleep and wake in mice. Biomed Eng Online. 2008;7:1–14.
3. Blume C, Santhi N, Schabus M. 'nparACT' package for R: A free software tool for the non-parametric analysis of actigraphy data. MethodsX. 2016;3:430.
4. Boumhaouad S, Makowicz EA, Choi S, Bouhaddou N, Balla J, Taghzouti K, et al. Regulation of Dopamine Release by Tonic Activity Patterns in the Striatal Brain Slice. ACS Chem Neurosci. 2025;16:310.
5. Schmitz Y, Schmauss C, Sulzer D. Altered dopamine release and uptake kinetics in mice lacking D2 receptors. The Journal of Neuroscience. 2002;22:8002–8009.
6. Martyniuk KM, Torres-Herraez A, Lowes DC, Rubinstein M, Labouesse MA, Kellendonk C. Dopamine D2Rs coordinate cue-evoked changes in striatal acetylcholine levels. Elife. 2022;11.
7. Ejdrup AL, Wellbourne-Wood J, Dreyer JK, Guldhammer N, Lycas MD, Gether U, et al. Within-Mice Comparison of Microdialysis and Fiber Photometry-Recorded Dopamine Biosensor during Amphetamine Response. ACS Chem Neurosci. 2023;14:1622–1630.
8. Lein ES, Hawrylycz MJ, Ao N, Ayres M, Bensinger A, Bernard A, et al. Genome-wide atlas of gene expression in the adult mouse brain. Nature 2006 445:7124. 2006;445:168–176.
